# Rethinking metagenome-assembled genome completeness: are we truly recovering complete genomes?

**DOI:** 10.3389/fmicb.2026.1884628

**Published:** 2026-07-06

**Authors:** Thierry Alexandre Pellegrinetti, Joshua Molligan, Lucas William Mendes, Alexandre Pedrinho, Edel Pérez-López

**Affiliations:** 1 Département de phytologie, Faculté des sciences de l‘agriculture et de l'alimentation, Université Laval, Québec, CA, Canada; 2 Centre de recherche et d'innovation sur les végétaux, Université Laval, Québec, CA, Canada; 3 Institute de Biologie Intégrative et des Systèmes, Université Laval, Québec, CA, Canada; 4 L'Institute EDS, Université Laval, Québec, CA, Canada; 5 Center for Nuclear Energy in Agriculture, University of São Paulo, São Paulo, Brazil; 6 School of Environmental and Conservation Sciences and the Harry Butler Institute, Murdoch University, Perth, WA, Australia

**Keywords:** genome completeness, genome quality estimation, genome-resolved metagenomics, metagenome assembled genome (MAG), microbial ecology

## 1 Introduction

Metagenome-assembled genomes (MAGs) have transformed microbiology by enabling genome-resolved characterization of uncultivated organisms directly from complex environmental sequence data. From the early reconstruction of acid mine drainage communities to large-scale global genome catalogs, MAGs have expanded the bacterial and archaeal tree of life and enabled ecological and evolutionary investigations at the genomic scale (Tyson et al., 2004; Parks et al., 2017; Nayfach et al., 2021). As MAG-based analyses have become increasingly widespread, however, genome quality scores are gradually repurposed in ways that extended beyond their original intent. In practice, MAGs classified as highly complete are frequently treated as close approximations of biological genomes, even when assembly fragmentation, strain heterogeneity, uneven coverage, and selective sequence loss remain considerable.

The issue lies not with completeness metrics themselves, but with the biological meaning often attached to them. Estimated completeness is commonly interpreted as broadly reflecting genomic recovery, even though the regions most difficult to reconstruct are frequently those most relevant to ecological and evolutionary inference. Regions associated with accessory and adaptive functions, including ribosomal operons, plasmids, genomic islands, and biosynthetic gene clusters (BGCs), are disproportionately vulnerable to assembly collapse, coverage filtering, and binning exclusion (Sczyrba et al., 2017; Shaiber and Eren, 2019; Maguire et al., 2020; Meziti et al., 2021). Consequently, a MAG may retain the genomic features required to satisfy quality thresholds while simultaneously losing regions associated with horizontal gene transfer (HGT), ecological specialization, host interaction, or adaptive metabolism.

This distinction becomes increasingly important as genome-resolved metagenomics moves beyond taxonomic cataloging toward ecological, functional, and evolutionary inference. A completeness score may summarize expected recovery of conserved genomic features, but it does not directly measure how much of the biologically informative genome has been reconstructed. As a result, downstream interpretations can carry a level of confidence that exceeds the underlying genomic evidence.

In this article, marker-based completeness refers to the inferred recovery of expected conserved markers or model-predicted genomic features used for quality assessment. Structural completeness refers to the physical reconstruction of genome architecture, including chromosome continuity, contig fragmentation, operons, repeats, plasmids, and mobile regions. Biological completeness refers to the recovery of the genome fraction needed to support a given ecological, functional, or evolutionary inference. These dimensions are related, but they are not equivalent.

## 2 Why completeness is not equivalent to biological recovery

Completeness metrics have become essential for standardizing genome quality assessment across large MAG datasets, and their utility for large-scale comparative microbiology is unquestionable (Parks et al., 2015; Bowers et al., 2017; Chklovski et al., 2023). However, completeness estimates remain indirect proxies for genome recovery rather than direct measurements of recovered biological content.

Marker placement introduces an additional source of bias because completeness markers are not uniformly distributed across microbial genomes. Many commonly used markers, including ribosomal proteins, occur within conserved operonic regions or tightly linked genomic neighborhoods (Wang et al., 2000; Tamames, 2001), meaning that completeness estimates may disproportionately reflect recovery of specific conserved genome fractions while providing limited information about flexible, mobile, or compositionally atypical regions. Similar interpretative limitations may also affect contamination estimates, particularly when repetitive or mobile sequences are inconsistently assembled, unevenly distributed across populations, or difficult to assign among closely related lineages (Orakov et al., 2021). In complex microbial communities, strain heterogeneity and horizontally shared genomic regions may additionally generate composite or consensus MAGs despite apparently low contamination estimates, further blurring the boundary between technical reconstruction quality and biological population structure.

Although completeness estimates can be generated using conserved marker genes, lineage-specific features, or machine-learning approaches, all of these strategies ultimately infer genome quality indirectly rather than directly measuring structural and compositional recovery. Repetitive operons may fail to assemble correctly, plasmids may be lost during binning, BGCs may span contig breaks, and adaptive accessory regions may be excluded due to atypical sequence composition or uneven coverage. In other words, a high completeness score does not necessarily imply proportional recovery of the genomic regions most relevant for ecological interpretation.

Importantly, the missing genomic fraction is unlikely to be biologically random. Regions repeatedly under-recovered in MAG workflows often include multi-copy elements that generate assembly ambiguity or atypical coverage, including rRNA operons, transposons, CRISPR arrays, plasmids, prophages and genomic islands, as well as compositionally atypical sequences that challenge k-mer- or codon-usage-based binning approaches, particularly on short contigs (Sczyrba et al., 2017; Nelson et al., 2020; Maguire et al., 2020). Mobile elements shared across closely related populations may additionally assemble into high-coverage consensus regions while remaining difficult to confidently assign to individual MAGs. Many of these loci encode functions central to microbial adaptation, competition, host interaction, antimicrobial resistance, HGT, or strain-level differentiation.

Conserved chromosomal regions are generally recovered more consistently than flexible or adaptive genome fractions, creating an important asymmetry in genome interpretation whereby completeness scores can remain high even when ecologically informative regions are substantially underrepresented. The issue therefore extends beyond missing sequence: different “regions” of the microbial genome are not equally recoverable, and their absence “can bias biological interpretation”.

Comparisons against isolate genomes show that MAGs with high estimated completeness may still lack population core genes, incorporate accessory genes incorrectly, or include sequence from unrelated taxa (Meziti et al., 2021), while composite MAGs can distort public genome resources and obscure population structure (Shaiber and Eren, 2019). Functional inference is similarly sensitive to incomplete or uneven genome recovery (Li and Yin, 2022; Eisenhofer et al., 2023). Together, these findings suggest that completeness scores are frequently interpreted as broader indicators of genomic representation than the recovered sequence can support.

Reduced symbiont genomes further illustrate why completeness should not be interpreted as a universal biological property. Organisms such as *Candidatus* Sulcia muelleri and *Candidatus* Nasuia deltocephalinicola maintain stable host-associated lifestyles despite possessing extremely reduced genomes that fall outside the assumptions embedded in conventional completeness models (McCutcheon and Moran, 2012). Similar problems can arise for large microbial groups with reduced or atypical genome architectures, including Candidate Phyla Radiation (CPR) bacteria and DPANN archaea, whose biology may not conform well to conventional completeness expectations (Brown et al., 2015; Castelle et al., 2015). In these cases, low estimated completeness reflects extreme genome reduction associated with host dependence rather than poor assembly quality or functional insufficiency. Conversely, environmentally reconstructed MAGs may achieve near-complete scores while lacking substantial accessory, adaptive, or strain-variable genome fractions. Completeness therefore reflects compatibility with expected models of genome architecture rather than a universal measure of biological sufficiency or ecological representativeness.

## 3 Comparative evidence from public genomes

We assessed bacterial MAGs from Genome Taxonomy Database (GTDB) release r226 (Parks et al., 2022) that were classified at species level and compared them with corresponding RefSeq reference genomes using average nucleotide identity (ANI) ≥ 95% as the species-level threshold. The dataset was manually curated to retain MAGs assigned to the species level, associated with a distinct reference genome, and derived from metagenomic reads, reducing the initial set from 584,382 to 28,356 MAGs. From this curated set, 14,000 MAGs were randomly selected using Python's random sample() function. Of these, 12,746 were successfully retrieved and included in subsequent analyses. Reference genomes were downloaded from RefSeq with NCBI-genome-download. ANI was calculated with fastANI (Jain et al., 2018), and only MAG-reference pairs with ANI ≥ 95% were retained, resulting in 9,960 MAGs and 1,216 reference genomes. MAGs were aligned against references with Minimap2 (Li, 2018), using ANI-adjusted presets such as asm5 for ANI ≥ 95%, and coverage statistics were computed with SAMtools (Li et al., 2009). Functional annotation was performed with Prokka (Seemann, 2014), and bidirectional protein similarity was assessed using DIAMOND BLASTp (Buchfink et al., 2021). Protein recovery was calculated as: Protein Hit Percentage = number of protein hits in MAG/total number of proteins in reference x 100. BGCs were identified with antiSMASH (Blin et al., 2021). Functional reconstruction used KofamScan (Aramaki et al., 2020) and KEGG-Decoder on 3,696 MAGs and 848 reference genomes randomly selected. Wilcoxon rank-sum tests were used to compare genomic features and pathway completeness, and environment-level comparisons were randomly balanced to limit unequal sample-size effects.

Because this analysis uses publicly available MAGs and reference genomes, the observed differences should be interpreted as comparative patterns rather than direct measurements of sequence loss caused only by MAG reconstruction. Reference genome availability, habitat representation, intra-species diversity, database curation, and differences among annotation or assembly pipelines can all influence MAG-reference contrasts. The analysis was therefore intended as an illustrative comparison of how estimated completeness relates to reference-based coverage and functional recovery, not as a formal benchmarking study or a precise estimate of reconstruction error.

**Figure 1:**
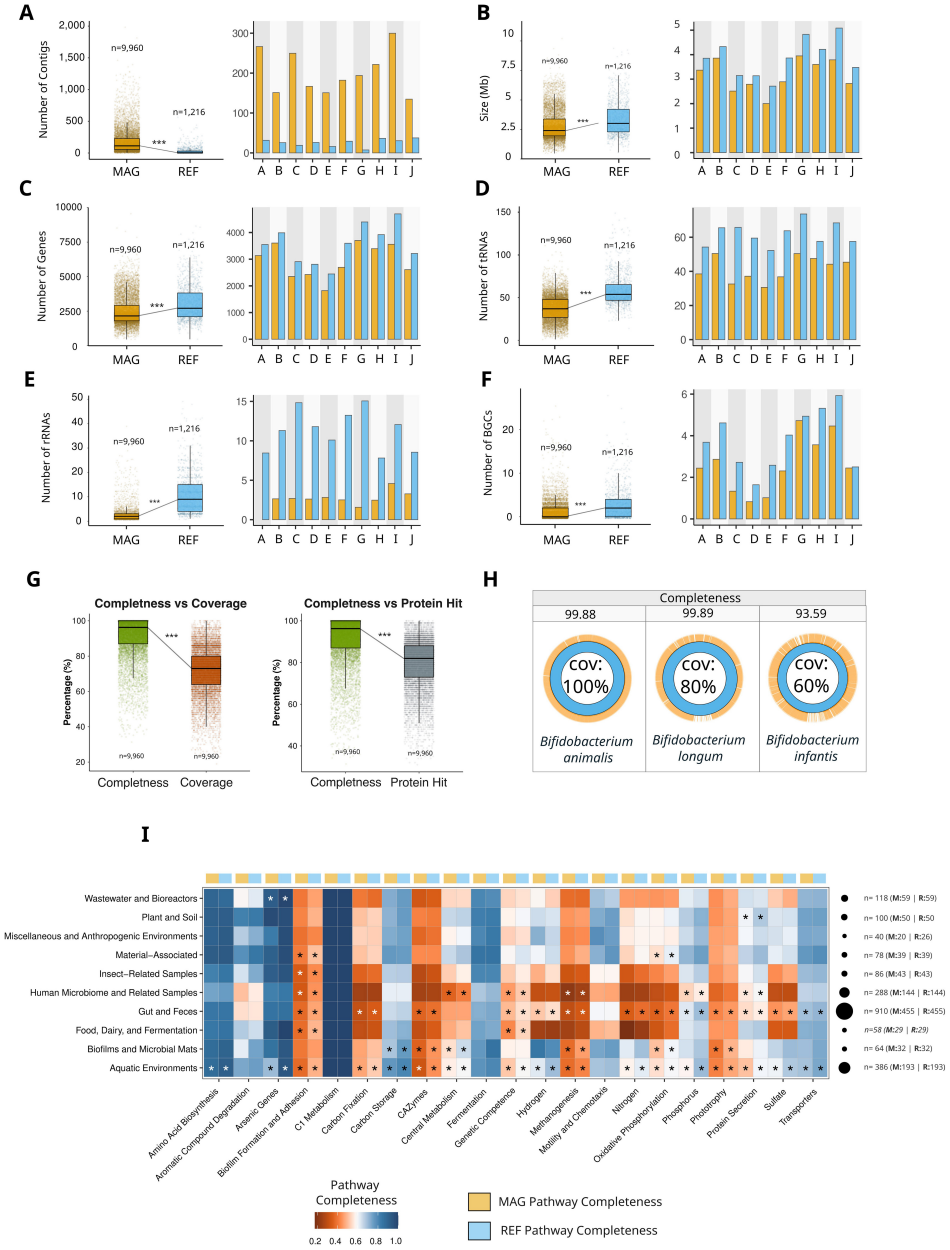
Structural and functional contrasts between metagenome-assembled genomes (MAGs) and reference genomes across diverse environments. Comparative analyses of 9,960 bacterial MAGs (ANI > 95%) retrieved from the Genome Taxonomy Database and matched against 1,216 reference genomes were used to illustrate differences in genomic architecture, gene recovery, and metabolic potential. Panels show comparisons of contig fragmentation **(A)**, genome size **(B)**, coding sequence abundance **(C)**, tRNA counts **(D)**, rRNA recovery **(E)**, biosynthetic gene clusters (BGCs); **(F)**, completeness estimates, reference-based genome coverage, and protein recovery rates **(G)**. Panel **(H)** highlights representative MAGs displaying high estimated completeness despite substantial variation in reference-genome coverage, illustrating potential discrepancies between completeness metrics and reference-based genomic representation. Panel **(I)** presents a pathway-level comparison between MAGs and corresponding reference genomes across environmental categories and metabolic functions; significance markers indicate statistically significant differences (*p* < 0.05). Side barplots in panels A-F summarize the environmental distribution of analyzed MAGs: A, Aquatic Environments; B, Biofilms and Microbial Mats; C, Food, Dairy and Fermentation; D, Gut and Feces; E, Human Microbiome and Related Samples; F, Insect-Associated Samples; G, Material-Associated Environments; H, Miscellaneous and Anthropogenic Environments; I, Plant and Soil; J, Wastewater and Bioreactors.

Across the dataset, MAGs tended to be more fragmented than their corresponding references and generally encoded fewer genes within smaller reconstructed genomes (Figures 1A-C). Although some reduction is expected given the inherent differences between isolate sequencing and metagenomic reconstruction, this pattern is consistent with a broader disconnect between estimated completeness and recovered biological content.

A similar pattern was observed for biologically informative loci. MAGs generally recovered fewer rRNAs, tRNAs, and BGCs than reference genomes (Figures 1D-F), consistent with the idea that regions difficult to reconstruct can be enriched in ecologically informative traits. Because these loci are frequently associated with secondary metabolism, microbial competition, signaling, host interaction, defense systems, and HGT, their selective under-recovery can bias ecological interpretation (Santana-Pereira et al., 2020; Hogle et al., 2022).

In the comparative dataset, reported completeness values were often high despite substantially lower reference coverage and protein hit percentages (Figures 1G, H), highlighting a disconnect between estimated completeness and broader genomic recovery. A MAG can therefore score as near complete while still lacking a nontrivial fraction of reference-aligned sequence or protein-coding content. Figure 1H illustrates this issue using selected *Bifidobacterium* MAGs. These MAGs were chosen as representative examples to illustrate the observed range of discrepancies between estimated completeness and reference-based coverage, not as universal or necessarily extreme cases. One MAG classified at 99.89% completeness recovered only ~80% of the reference genome, whereas another classified at 93.59% completeness recovered ~60%. This pattern suggests that inferred completeness, recovered sequence content, and ecological interpretability are related but non-equivalent dimensions of genome quality.

Pathway-level comparisons were consistent with a similar pattern (Figure 1I). Some metabolic categories were represented across MAGs and references, whereas others showed substantial attenuation. Importantly, these differences were not uniformly distributed across functions, suggesting that genome incompleteness may affect ecological inference directionally rather than randomly.

## 4 Consequences for genome-resolved ecology

Once completeness scores are interpreted as proxies for whole-genome recovery, downstream analyses can become susceptible to inherited overconfidence. In comparative genomics, technical absence may be misinterpreted as evolutionary gene loss. In pangenomics, under-recovery of flexible genome regions can artificially compress accessory repertoires and inflate apparent core genome conservation (Li and Yin, 2022). In microbial ecology, incomplete reconstruction may underestimate secondary metabolism, mobile element burden, defense capacity, strain-level differentiation, or adaptive potential.

These distortions are likely most pronounced for traits concentrated outside conserved chromosomal cores. Plasmids, genomic islands, phage-associated loci, secretion systems, defense islands, and strain-variable regions disproportionately encode antimicrobial resistance, host interaction, microbial competition, niche specialization, and HGT (Maguire et al., 2020; Abdulkadir et al., 2024). A MAG may therefore provide robust evidence for conserved metabolism while simultaneously offering limited representation of adaptive ecology.

Findings from Eisenhofer et al. (2023) demonstrated that metagenomic functional profiling changes substantially with genome completeness, indicating that pathway-level conclusions may inherit reconstruction biases from missing genomic content. Gene absence in a MAG should therefore be interpreted differently from absence in a closed isolate genome, particularly for repetitive, mobile, accessory, or low-abundance regions.

Long-read sequencing, hybrid assemblies, assembly-graph approaches, single-cell genomics, and metatranscriptomics are already improving recovery of genomic regions traditionally lost in short-read MAG workflows (Quince et al., 2021; Singleton et al., 2021; Sanchez-Navarro et al., 2022). Comparisons between short-read and long-read or hybrid metagenomic workflows indicate that sequencing strategy can affect contiguity and recovery of repetitive regions, mobile elements, and BGCs, although long-read approaches do not eliminate assembly, binning, strain-resolution, or reference database limitations. Nevertheless, no reconstruction strategy removes the need to distinguish between estimated genome quality and recovered biological content.

## 5 Discussion

Completeness estimates were originally designed as operational summaries for genome quality control, yet they are increasingly interpreted as broader indicators of biological reconstruction than the recovered sequence itself can support (Parks et al., 2015; Bowers et al., 2017; Chklovski et al., 2023). As genome-resolved metagenomics expands from taxonomic cataloging toward ecological and evolutionary inference, this interpretative shift becomes increasingly consequential.

The central issue is not the usefulness of MAGs or the metrics used to assess their quality. Their value for studying uncultivated microbial diversity is unquestionable (Parks et al., 2017; Nayfach et al., 2021). Rather, the problem emerges when completeness scores are implicitly treated as proxies for whole-genome recovery, despite the uneven recoverability of microbial genome architecture. Multiple independent studies, together with our comparative analyses, indicate that repetitive, mobile, accessory, and strain-variable regions remain disproportionately vulnerable to assembly fragmentation and binning exclusion (Shaiber and Eren, 2019; Maguire et al., 2020; Meziti et al., 2021).

This distinction is particularly important because these regions may frequently encode traits central to microbial adaptation and ecological interpretation, including HGT, secondary metabolism, host interaction, antimicrobial resistance, and niche specialization (Maguire et al., 2020; Abdulkadir et al., 2024). As a result, absence in a MAG cannot necessarily be interpreted as biological absence in the organism itself, particularly when conclusions depend on flexible or accessory genome content.

The issue therefore extends beyond technical genome quality assessment. Biological completeness, structural completeness, and marker-based completeness are related but fundamentally non-equivalent concepts. Reduced endosymbiont genomes illustrate this clearly, as organisms with highly streamlined genomes may fall outside conventional completeness expectations while remaining biologically stable (McCutcheon and Moran, 2012). Conversely, environmentally reconstructed MAGs may satisfy high-quality thresholds while still lacking substantial adaptive genome fractions.

Completeness metrics remain indispensable because they make genome-resolved metagenomics scalable. However, their biological interpretation should remain proportional to the type of inference being made. Conserved metabolic reconstruction may be reasonably supported by high completeness estimates, whereas conclusions involving adaptive ecology, strain-level variation, mobile elements, or accessory metabolism require additional structural and comparative evidence. Genome-resolved microbiology does not require less confidence in MAGs, but greater precision regarding what that confidence actually refers to.
